# 

**Published:** 1924-06

**Authors:** 


					New Series. Vol. III. No. 33 rfc.?1')- June, 1924. Monthly, Price 6d. (P0S7TJREE)

				

